# Association between hatching status and pregnancy outcomes in single blastocyst transfers: a retrospective cohort analysis

**DOI:** 10.1007/s10815-025-03450-4

**Published:** 2025-03-28

**Authors:** Weimin Yang, Qingkai Wang, Bin Zhang, Ross Ka-Kit Leung, Kai Deng, Shuangshuang Geng, Jinfeng Xu, Yu Qiao, Hui Gao, Dongchuan Li, Liyi Cai

**Affiliations:** 1 Hebei Maternity Hospital, Shijiazhuang City, Hebei Province, China; 2 Shi Jiazhuang Technology Innovation Center of Precision Prevention and Control of Birth Defects, Shijiazhuang City, Hebei Province, China

**Keywords:** Single blastocyst transfer, Assisted hatching, Hatching status, Blastocyst morphology, Embryo selection

## Abstract

**Objective:**

To examine the potential association between blastocyst hatching status and pregnancy outcomes following single blastocyst transfer.

**Methods:**

This is a retrospective cohort. We screened all frozen-thawed single blastocyst transfer cycles from January 1, 2020, to April 30, 2022, at the authors’ center. The hatching status was graded into four categories: unhatched, early hatching (hatched portion < the diameter of zona pellucida), late hatching (hatched portion > the diameter of zona pellucida), and fully hatched. Multivariate logistic regression was used to examine the association between hatching status and pregnancy outcomes (clinical pregnancy and live birth).

**Results:**

The final analysis included 906 cycles. The hatching status was unhatched in 116 cycles, early hatching in 556 cycles, late hatching in 197 cycles, and fully hatched in 37 cycles. The clinical pregnancy rate was 54.3%, 63.3%, 74.6%, and 54.1%, respectively (*p* = 0.001) in the unhatched, early-hatching, late-hatching, and fully hatched groups, respectively. The live birth rate was 39.7%, 51.6%, 58.3%, and 40.5%, respectively (*p* = 0.008). In pairwise comparisons, the late-hatching group had significantly higher rates of clinical pregnancy and live birth versus the unhatched category (*p* = 0.001 and *p* = 0.008, respectively). In multivariate logistic regression analysis, embryo hatching status, the duration until blastocyst formation, the grading of blastocyst cells, and the thickness of the endometrium were associated with clinical pregnancy and live birth.

**Conclusion:**

After adjusting for confounding factors, late-hatching status of the blastocysts was associated with a higher rate of clinical pregnancy and live birth.

**Supplementary Information:**

The online version contains supplementary material available at 10.1007/s10815-025-03450-4.

## Introduction

The evolution of assisted reproductive technologies has made single blastocyst transfer (SBT) a pivotal strategy to enhance the efficacy of in vitro fertilization-embryo transfer (IVF-ET) cycles [1, 2]. Numerous factors influence the success of single embryo transfer, among which advancing maternal age significantly increases the incidence of chromosomal abnormalities in embryos, negatively impacting pregnancy rates [3]. This increase is primarily attributed to the diminished precision of chromosomal segregation in aging oocytes, hereby elevating the likelihood of aneuploidy. Furthermore, an optimal endometrial thickness, ranging from 7 to 14 mm, is intricately associated with increased implantation rates and the successful establishment of pregnancy [4, 5]. Deviations from this range, whether too thin or too thick, can hinder effective embryo implantation and subsequently affect pregnancy outcomes. Therefore, a thorough analysis of these influencing factors is crucial for optimizing the clinical effectiveness of SBT.

Empirical evidence suggests that the transfer of a single high-quality blastocyst reduces the risks of preterm birth and multiple pregnancies [6, 7]. In addition to the quality of the inner cell mass (ICM) and trophectoderm (TE), the degree of blastocyst expansion reflects the embryo’s developmental trajectory and physiological integrity and thus affects the implantation potential [8]. In a study of 1488 single frozen-thawed SBT cycles by Goto et al. [9], a higher level of expansion was associated with improved pregnancy outcomes, including increased implantation success and live births. Research has also shown that euploid human blastocysts showed higher expansion grades and shorter time to start blastulation, expansion, and hatching [10].

Prolonged exposure to artificial conditions could impair the implantation capacity of human oocytes and embryos. A phenomenon known as “zona pellucida (ZP) hardening” is considered one of the causes of embryo implantation failure after in vitro culture. This phenomenon can be addressed through laser-assisted hatching (LAH) [11–14]. Hiraoka et al. demonstrated that LAH with 50% zona pellucida opening significantly increases clinical pregnancy, implantation, and live birth rates in women with recurrent implantation failures [15].

We conducted a retrospective analysis to examine whether the hatching status of the blastocyst prior to transfer is associated with pregnancy outcomes in frozen-thawed embryo transfer (FET) cycles.

## Methods

### Study population

**Fig. 1:**
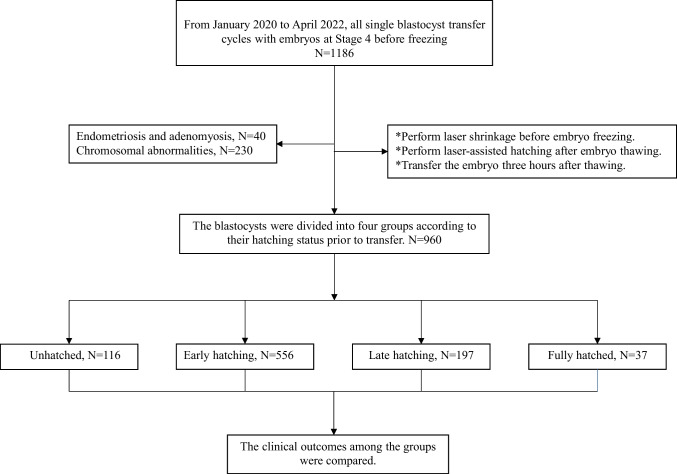
Flow diagram illustrating the selection process for study participants, including exclusion criteria, and the categorization of blastocyst hatching status before embryo transfer

**Fig. 2:**
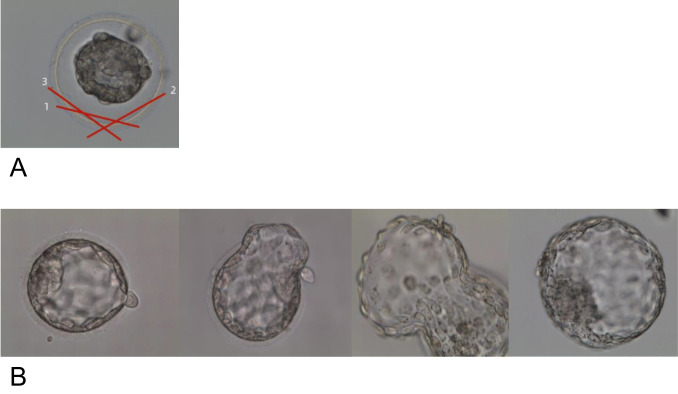
Laser-assisted hatching method and pre-embryo transfer hatching status. **A** A schematic representation of the laser-assisted hatching procedure, where 10–15 laser beams are used to remove a small portion of the zona pellucida (ZP) area within the empty perivitelline space. This excision is performed three times to ensure the removal of this section of the zona pellucida, with numerical indicators denoting the order of steps. **B** Sequential arrangement of groups categorized by hatching status, displayed from left to right as follows: unhatched, early hatching, late hatching, and fully hatched

A retrospective analysis was conducted on patients who underwent SBT between January 2020 and April 2022 at the authors’ center. Patients with endometriosis, adenomyosis, or chromosomal abnormalities were excluded (as shown in Fig. 1). The blastocysts were all at stage 4 before freezing and were classified into four categories based on their hatching status: unhatched, early hatching (hatched portion < the diameter of the zona pellucida), late hatching (hatched portion > the diameter of the zona pellucida), and fully hatched (as shown in Fig. 2).

### Ovulation stimulation and embryo culture

Ovulation was induced using a standard antagonist protocol consisting of early follicular phase long protocols and progestin-primed ovarian stimulation (PPOS). Gonadotropin dosage was adjusted according to patient response. Trigger shots consisted of 5000 to 8000 IU human chorionic gonadotrophin (hCG) or 0.1 mg gonadotropin-releasing hormone agonist (GnRHa) with 4000 IU hCG, followed by egg retrieval 37 h later under transvaginal ultrasound guidance, followed by IVF or intracytoplasmic sperm injection (ICSI). On the morning of the first day after egg retrieval at 7:30 am, the presence of pronuclei was observed. All embryos were fertilized and cultured using a commercial culture medium (Vitrolife, Sweden, Catalog Nos.: 10086, 10,127, 10,131). To maintain a stable culture environment, 100% mineral oil (SAGE Laboratories, Denmark, Product No.: ART-4008) was overlaid on the culture dishes. The culture conditions were strictly controlled at 37 °C, with a constant atmosphere of 5% carbon dioxide (CO₂) and 5% oxygen (O₂), ensuring optimal conditions for embryo development.

### Morphological assessment of blastocysts

Prior to freezing, blastocysts were assessed using the Gardner and Schoolcraft system [16, 17]. The blastocysts were graded according to the number of ICM and TE cells and their morphology: grade A indicates a high number of tightly packed ICM cells and a substantial TE cell layer forming a cohesive epithelium, grade B indicates a lower number of loosely arranged ICM cells and a sparse TE cell layer, and grade C denotes very few or nearly absent ICM cells, with TE cells being minimal or scattered. D5/D6 blastocysts classified as grades AA, AB, BA, or BB are considered high quality. Blastocysts graded as AC, BC, CA, or CB, along with the aforementioned high-quality blastocysts were considered usable.

### Vitrification and thawing of blastocysts

Embryos vitrification and thawing were performed using a commercial kit from Kitazato (Shizuoka, Japan), as previously described [18].

### Blastocyst laser-induced collapse and assisted incubation

Prior to vitrification, blastocysts were aligned for laser treatment, targeting the trophoblast cell junction opposite the inner cell mass. A precise laser pulse was applied, adjusting the emission duration to ensure a controlled opening. Following the procedure, blastocysts were incubated at 37 °C in a 6% CO₂ environment for 10 min to allow complete collapse of the blastocoel cavity before proceeding with vitrification. All blastocysts included in this study were at stage 4. Laser-assisted hatching for all blastocysts was performed post-thawing using the side opposite to the hatching site in the inner cell mass where the zona pellucida appeared wider or presented more fragments. Approximately one-fourth of the zona pellucida was removed using laser ablation, with the procedures shown in Fig. 2A. Blastocysts were then incubated under the same conditions for 3 h before transfer, with photographic documentation taken for each specimen.

### Endometrial preparation

Endometrial preparation was conducted as described below. The natural cycle involves monitoring follicle growth and endometrial thickness via transvaginal ultrasound. Ovulation was estimated with follicle diameter ≥ 14 mm and serum luteinizing hormone (LH), estradiol (E2), and progesterone levels. If endometrial thickness was ≥ 8 mm and LH level was ≥ 25 mIU/ml, ovulation was triggered with either hCG (8000–10,000 IU) or Ovidrel (250 mg). Post-ovulation, 20–40 mg progesterone was administered intramuscularly. Blastocyst transfer was scheduled 5 days post-ovulation, with luteal support initiated on the day of transfer (90 mg progesterone gel and 20 mg dydrogesterone per day). For the ovulation induction cycle, letrozole was administered orally at 2.5–5.0 mg daily from days 3–5 of the menstrual cycle for 5 consecutive days. Ultrasound monitoring began on days 10–12. Subsequent steps were identical to that in the natural cycle. For the artificial cycle, E2 was started on day 3 of the menstrual cycle at a dose of 6–8 mg/day. Endometrial thickness was monitored using ultrasound, and estradiol and progesterone levels were measured to guide dosage adjustments. After at least 10 days of medication, if the endometrial thickness reaches ≥ 8 mm, progesterone (40–60 mg) was injected to induce endometrial transformation. Blastocyst transfer was performed 5 days later, with luteal support starting on the day of transfer. For the down-regulation artificial cycle, a long-acting GnRH agonist was injected subcutaneously on days 2–3 of the menstrual cycle. After 28 days, estradiol valerate was administered orally, using a protocol identical to the artificial cycle.

### Statistical methods

Normally distributed continuous variables were analyzed using one-way analysis of variance (ANOVA) and expressed as mean ± standard deviation. Non-normally distributed continuous variables were analyzed using the Kruskal–Wallis *H* test and expressed as the median and interquartile range (Q1, Q3). Categorical variables were evaluated using the Pearson ×^2^ test. Multivariate logistic regression analysis was conducted to identify factors that were associated with clinical pregnancy and live birth. Factors included in the multivariate regression as independent variables included demographic variables and the results of statistical comparison between groups using a criterion of *p* < 0.1. The final model included the age of women, endometrial thickness, AMH, follicle-stimulating hormone (FSH), the number of days to blastocyst formation, hatching status before embryo transplantation, and the grading of blastocyst cells. Results of the multivariate regression are shown as odds ratio (OR) and 95% confidence interval (CI). A *p*-value of < 0.05 was considered statistically significant. All statistical analyses were conducted using SPSS version 25.0.

## Results

### Cohort characteristics

The final analysis included 906 cycles (Table 1). The hatching status was unhatched in 116 cycles, early hatching in 556 cycles, late hatching in 197 cycles, and fully hatched in 37 cycles. The four groups did not difference in age, body mass index (BMI), duration and type of infertility, endometrial thickness, or baseline FSH. Significant differences were observed in baseline LH (*p* = 0.038) and baseline E2 (*p* = 0.004), the duration of blastocyst culture (*p* < 0.001), and blastocyst quality (*p* < 0.001).

**Table 1:** Comparison of the general clinical characteristics of patients in the four groups

Variable	Unhatched	Early hatching	Late hatching	Fully hatched	*p*
No. of patients	116	556	197	37	
Age of women (years)	32.23 ± 4.84	31.96 ± 4.44	31.68 ± 4.49	31.16 ± 3.88	0.526
BMI (kg/m^2^）	24.90 ± 4.66	24.82 ± 4.34	24.26 ± 4.32	25.40 ± 4.77	0.319
Duration of infertility (years)	3.00 (1.00, 4.00)	3.00 (1.00, 5.00)	3.00 (1.00, 5.00)	2.00 (1.00, 5.00)	0.767
Endometrial thickness (mm)	9.76 ± 1.86	9.66 ± 1.77	9.59 ± 1.82	9.46 ± 1.93	0.799
Type of Infertility					0.881
Primary	48 (41.38)	249 (44.78)	84 (42.64)	17 (45.95)	
Secondary	68 (58.62)	307 (55.22)	113 (57.36)	20 (54.05)	
Basal hormone profile					
FSH (mIU/mL)	6.79 (5.79, 8.56)	6.86 (5.95, 8.02)	7.20 (6.17, 8.38)	7.35 (6.01, 8.09)	0.462
LH (mIU/mL)	4.64 (3.63, 6.48)	4.56 (3.19, 6.51)	4.59 (3.29, 6.78)	3.47 (2.49, 5.12)	0.038
E2 (pg/mL)	32.64 (24.84, 47.33)	38.02 (28.86, 52.00)	40.00 (30.39, 51.12)	47.00 (32.00, 54.00)	0.004
AMH (ng/mL)	4.46 (3.01, 7.46)	4.302 (2.49, 7.10)	4.17 (2.29, 6.62)	3.85 (2.12, 5.04)	0.204
Day of blastocyst, *n* (%)					< .001
Day 5	90 (77.59)	456 (82.01)	152 (77.16)	19 (51.35)	
Day 6	26 (22.41)	100 (17.99)	45 (22.84)	18 (48.65)	
Blastocyst quality					< .001
Poor	23 (19.83)	82 (14.75)	25 (12.69)	14 (37.84)	
High quality	93 (80.17)	474 (85.25)	172 (87.31)	23 (62.16)	

### Pregnancy outcomes

The rate of biochemical pregnancy was 70.7%, 80.9%, 82.2%, and 75.7% in the unhatched, early-hatching, late-hatching, and fully hatched groups, respectively (*p* = 0.058). The rate of clinical pregnancy was 54.3%, 63.3%, 74.6%, and 54.1%, respectively (*p* = 0.001). The rate of live births was 39.7%, 51.6%, 58.3%, and 40.5%, respectively (*p* = 0.008). In the pairwise comparisons, the late-hatching group had a higher rate of clinical pregnancy and live birth versus the unhatched category (*p* = 0.001 and *p* = 0.008, respectively) (Table 2). Among all high-quality embryos, the late-hatching group showed better clinical pregnancy rates than the other three groups (*p* = 0.045) (Supplementary Table 1). In hormone replacement cycles, the late-hatching group had the highest clinical pregnancy and live birth rates among the four groups (Supplementary Table 5). Among all day 5 embryos, there was a statistically non-significant trend for higher clinical pregnancy and live birth rates in the late-hatching group (Supplementary Table 6). In IVF cycles, the late-hatching group also had the highest clinical pregnancy and live birth rates (Supplementary Table 7).

**Table 2:** Clinical outcomes

Variables	Unhatched	Early hatching	Late hatching	Fully hatched	*p*
	*n* = 116	*n* = 556	*n* = 197	*n* = 37	
Biochemical pregnancy, *n* (%)	82 (70.69)	450 (80.94)	162 (82.23)	28 (75.68)	0.058
Clinical pregnancy, *n* (%)	63 (54.31)^a^	352 (63.31)	147 (74.62)^b^	20 (54.05)	0.001
Early miscarriage, *n* (%)	12 (19.05)	49 (13.92)	23 (15.65)	4 (20.00)	0.670
Live birth, *n* (%)	46 (39.66)^a^	287 (51.62)	115 (58.38)^b^	15 (40.54)	0.008
Note: Values in the same column with different lowercase letter superscripts are significantly different (*p *< 0.05)					

### Factors associated with clinical pregnancy

The clinical pregnancy rate was 68.0% for D5 embryos versus 48.6% for D6 embryos (*p* < 0.001) (Supplementary Table 1). Grade C ICM was associated with a lower clinical pregnancy rate compared to Grades A and B (C 38.0%, A 74.1%, B 60.3%; C vs. A *p* < 0.05, C vs. B *p* < 0.05). Grade C TE was also associated with a lower clinical pregnancy rate (C 38.9%, A 74.4%, B 65.0%; C vs. A *p* < 0.05, C vs. B *p* < 0.05) (Table 3). In multivariate regression analysis, late hatching was associated with a higher rate of clinical pregnancy after adjusting for age, BMI, AMH level, day of embryo transfer, and embryo quality (adjusted OR 2.417, 95% CI 1.492–3.915) (Table 3). Subgroup analysis showed that, in both the < 35-year subgroup and the ≥ 35-year subgroup, the late-hatching group had significantly higher clinical pregnancy rates compared to the unhatched group (76.0% vs. 61.7%, adjusted OR 2.109 [1.142–3.896]; 70.6% vs. 38.9%, adjusted OR 3.964 [1.466–10.732]). In the ≥ 35-year subgroup, the late-hatching group had a significantly higher clinical pregnancy rate compared to the unhatched group (70.6% vs. 38.9%, adjusted OR 3.964 [1.466–10.732]). Regarding embryo quality, the overall subgroup analysis did not show significant interaction (*p* = 0.198) (Supplementary Table 4).

**Table 3:** Multivariate regression analysis of clinical pregnancy

	β	*p*	OR (95% Cl)
Age of women, years	− 0.043	0.020	0.957 (0.923–0.993)
Endometrial thickness	0.199	0.001	1.220 (1.120–1.330)
ICM			
C			ref
A	1.215	0.004	3.372 (1.482–7.669)
B	0.875	0.027	2.398 (1.103–5.217)
TE			
C			ref
A	0.866	0.005	2.377 (1.298–4.352)
B	0.706	0.003	2.025 (1.274–3.220)
Day of blastocyst			
Day 6			ref
Day 5	0.570	0.003	1.768 (1.210–2.584)
Hatching status			
Unhatched			ref
Early hatching	0.284	0.209	1.328 (0.853–2.067)
Late hatching	0.835	0.002	2.305 (1.362–3.901)
Fully hatched	0.344	0.410	1.410 (0.623–3.191)

Note: Adjusted with female age, AMH, baseline FSH, endometrium thickness, transfer day, hatching status before embryo transplantation, and the grading of blastocyst cells

*ICM* inner cell mass, *TE* trophectoderm, *OR* adjusted odds ratio, *CI* confidence interval, *ref* reference

### Factors associated with live birth

The live birth rate was 54.5% for D5 embryos versus 35.0% for D6 embryos (*p* < 0.001) (Supplementary Table 3). Grade C inner cell mass was associated with a lower live birth rate compared to Grades A and B (C 26.0%, A 59.5%, B 47.1%; C vs. A *p* < 0.05, C vs. B *p* < 0.05). Grade C trophectoderm cells were also associated with lower live birth rates (C 30.6%, A 64.6%, B 49.8%; C vs. A *p* < 0.05, C vs. B *p* < 0.05) (Table 4). In multivariate regression analysis, late hatching (adjusted OR 2.053, 95% CI 1.281–3.291) and early hatching (adjusted OR 1.607, 95% CI 1.060–2.436) were associated with higher live birth rate after adjusting for potential confounders (Table 4).

**Table 4:** Multivariate regression analysis of live birth

	β	*p*	OR (95% Cl)
Age of women, years	− 0.059	0.001	0.943 (0.910–0.977)
Endometrial thickness	0.226	0.001	1.253 (1.154–1.360)
ICM			
C			ref
A	0.858	0.042	2.435 (1.034–5.580)
B	0.646	0.106	1.944 (0.849–4.451)
TE			
C			ref
A	0.748	0.013	2.114 (1.170–3.819)
B	0.390	0.107	1.476 (0.920–2.370)
Day of blastocyst			
Day 6			ref
Day 5	0.557	0.004	1.745 (1.196–2.548)
Hatching status			
Unhatched			ref
Early hatching	0.438	0.051	1.550 (0.998–2.406)
Late hatching	0.725	0.005	2.066 (1.249–3.416)
Fully hatched	0.331	0.424	1.393 (0.618–3.140)

Note: Adjusted with female age, AMH, baseline FSH, endometrium thickness, transfer day, hatching status before embryo transplantation, and the grading of blastocyst cells

*ICM* inner cell mass, *TE* trophectoderm, *OR* adjusted odds ratio, *CI* confidence interval, *ref* reference

## Discussions

LAH is an assisted reproductive technology that has been widely applied in recent years. It provides a gentler method for embryo hatching, allowing embryos to hatch without damage, thus increasing their chances of implantation and pregnancy success. Multiple studies support the effectiveness of laser-assisted hatching, particularly in cases of thick zona pellucida or frozen embryo transfers, where it helps improve hatching and implantation rates. A study by Du et al. [19] suggested that the blastocyst expansion stage (from stage 3 to stage 6) before transfer is a significant predictor of clinical pregnancy and live birth.

In the current study, the late-hatching status was associated with a higher biochemical pregnancy rate (82.3%), clinical pregnancy rate (74.6%), and live birth rate (58.4%) compared to other groups. A higher rate of pregnancy outcomes in the late-hatching group versus the unhatched and early-hatching groups may reflect the improved developmental potential of the blastocysts. The high implantation success rate of expanded blastocysts may be due to the fact that expansion provides more space for the blastocyst, enhancing the interaction between cells and the surrounding environment, thus helping the blastocyst better adhere to and implant into the endometrium [20].

Analysis of the pregnancy outcomes in high-quality blastocysts suggested a statistically non-significant trend for higher clinical pregnancy and live birth rates in the late-hatching group. The analysis of pregnancy outcomes for day-5 blastocysts also showed a statistically non-significant trend of better clinical outcomes in the late-hatching group compared to the other three groups. This may be due to the small variability among high-quality blastocysts and insufficient sample size. Similar to a study by Razi et al. [21], a significant association between hatching status and both clinical pregnancy and live birth rates was observed in IVF cycles, but not in ICSI cycles in the current study. We believe that larger sample sizes in future studies are needed to further validate these findings and explore potential reasons, such as whether the drilling effect of the zona in ICSI cycles might already influence the hatching process, thereby affecting the relationship between hatching status and clinical outcomes. A previous study by Vajta et al. [22] suggested that complete removal of the zona pellucida might improve implantation rates by reducing the energy expenditure of embryos. In the current study, pregnancy outcomes in the fully hatched group were poorer than in the late-hatching group. We speculate that the complete removal of the zona pellucida leaves the blastocyst vulnerable to environmental damage during handling, especially in blastocysts that have reached stage 6.

Consistently with previous studies [23–25], multivariate regression analysis in the current study indicated that live birth was associated with age, BMI, endometrial thickness on the day of transfer, and embryo quality. A previous study showed embryos with an ICM grade of A have the highest clinical pregnancy and live birth rates [26]. Several recent studies, however, suggested that a higher ICM grade is associated with a lower miscarriage rate and higher birth weight [27, 28]. The current study did not find an association between ICM grading and live birth, which may be due to the small sample size of blastocysts with an ICM grade of C. Day-5 blastocysts are typically associated with higher implantation potential, whereas day-6 blastocysts may show reduced efficiency due to delayed developmental milestones [29]. Consistently, the current study found better clinical outcomes with day-5 versus day-6 blastocysts.

Subgroup analysis showed a significant interaction between the hatching status and female age, indicating that female age may influence the observed effect. In both the < 35-year and ≥ 35-year subgroups, the late-hatching group showed a significantly higher clinical pregnancy rate compared to the unhatched group, but the magnitude of the difference was larger in the ≥ 35-year subgroup. With regards to embryo quality, the overall interaction was not significant (*p* = 0.198), and the late-hatching group exhibited higher clinical pregnancy rates in both the non-high-quality and high-quality embryo subgroups (Supplementary Table 4). Late-hatching embryos showed a clear advantage in improving clinical pregnancy rates, especially in older women (≥ 35 years), regardless of embryo quality. Consistent with the findings by Wei et al. [30], the clinical outcomes in the late-hatching group in the current study were significantly better than in other groups in hormone replacement cycles.

This study has several limitations. First, the sample size for the fully hatched group was small, primarily due to the short interval of approximately 3 h between thawing and transfer, which resulted in a limited number of blastocysts reaching the expanded blastocyst stage. Additionally, in this study, the hatching status of blastocysts was classified based on visual inspection of the pre-transfer images, which may introduce subjective bias. Using more precise measurement techniques, such as continuous variable assessment instead of categorizing blastocyst hatching into four discrete groups, could provide more detailed data and enhance the power of statistical analyses. Furthermore, this study was a single-center retrospective study with limited control of confounding variables. Future multi-center prospective studies may help improve the generalizability of the findings and increase the robustness of the results. In summary, the current study suggests that late-hatching status that exceeds the original zona pellucida diameter is associated with improved pregnancy outcomes in single blastocyst transfer cycles. Future research should further optimize LAH techniques, particularly in different patient populations, such as those undergoing frozen embryo transfers, patients with repeated IVF failures, and older patients. Additionally, the safety of complete zona pellucida removal and its specific impact on blastocyst hatching and implantation processes should be explored. Further evaluation of the effects of ICM grading on embryo development and pregnancy outcomes, particularly through large-scale clinical data, is also necessary to provide more reliable guidance for clinical practice.

## Supplementary Information

Below is the link to the electronic supplementary material.

Supplementary file1 (DOCX 18 KB)

Supplementary file2 (DOCX 22 KB)

Supplementary file3 (DOCX 20 KB)

Supplementary file4 (DOCX 19 KB)

Supplementary file5 (DOCX 20 KB)

Supplementary file6 (DOCX 17 KB)

Supplementary file7 (DOCX 18 KB)

## Data Availability

The datasets used and analyzed in this study are available from the corresponding author upon reasonable request.
