# Supplementary material for: Association between hatching status and pregnancy outcomes in single blastocyst transfers: a retrospective cohort analysis
Source: J Assist Reprod Genet. 2025 Mar 28;42(5):1707–15. doi: 10.1007/s10815-025-03450-4 (PMC12167215; doi:10.1007/s10815-025-03450-4)
Supplement: Supplementary file 1 — Supplementary file1 (DOCX 18 KB) [file 10815_2025_3450_MOESM1_ESM.docx]

| Supplementary table 1 Clinical outcomes among the four groups with the same embryo quality | | | | | |
| --- | --- | --- | --- | --- | --- |
|  | Poor embryos | | | | *P* |
| Clinical Outcome | Unhatched  n=23 | Early hatching  n=82 | Late hatching  n=25 | Fully hatched  n=14 |  |
| Positive hCG | 11 (47.83%) | 58 (70.73%) | 22 (88.00%) | 9 (64.29%) | 0.025 |
| Clinical pregnancy | 4 (17.39%) | 36 (43.90%) | 14 (56.00%) | 5 (35.71%) | 0.045 |
| Early miscarriage | 1 (25.00) | 4 (11.11) | 5 (35.71) | 1 (20.00) | 0.166 |
| Live birth | 2 (8.70) | 32 (39.02) | 9 (36.00) | 4 (28.57) | 0.051 |
|  | High-quality embryos | | | | *P* |
| Clinical Outcome | Unhatched  n=93 | Early hatching  n=474 | Late hatching  n=172 | Fully hatched  n=23 |  |
| Positive hCG | 71 (76.34) | 392 (82.70) | 140 (81.40) | 19 (82.61) | 0.549 |
| Clinical pregnancy | 59 (63.44) | 316 (66.67) | 133 (77.33) | 15 (65.22) | 0.042 |
| Early miscarriage | 11 (18.64) | 45 (14.24) | 18 (13.53) | 3 (20.00) | 0.738 |
| Live birth | 44 (47.31) | 255 (53.80) | 106 (61.63) | 11 (47.83) | 0.113 |
