# Supplementary material for: Association between hatching status and pregnancy outcomes in single blastocyst transfers: a retrospective cohort analysis
Source: J Assist Reprod Genet. 2025 Mar 28;42(5):1707–15. doi: 10.1007/s10815-025-03450-4 (PMC12167215; doi:10.1007/s10815-025-03450-4)
Supplement: Supplementary file 2 — Supplementary file2 (DOCX 22 KB) [file 10815_2025_3450_MOESM2_ESM.docx]

| Supplementary table 2 Analysis of factors related to clinical pregnancy | | | |
| --- | --- | --- | --- |
| Variables | Non-clinical pregnancy  (n = 324) | Clinical pregnancy  (n = 582) | *P* |
|  |  |  |  |
| Age of women (years) | 32.65 ± 4.69 | 31.48 ± 4.31 | <.001 |
| BMI (kg/m²） | 23.90 (21.63, 27.39) | 24.05 (21.48, 27.34) | 0.756 |
| Endometrial thickness (mm） | 9.26 ± 1.73 | 9.87 ± 1.80 | <.001 |
| Duration of infertility (years) | 3.00 (1.00, 5.00) | 3.00 (1.00, 5.00) | 0.729 |
| Basal hormone profile |  |  |  |
| FSH (mIU/mL） | 7.13 (6.19, 8.55) | 6.86 (5.81, 7.96) | 0.011 |
| LH (mIU/mL） | 4.39 (3.14, 6.34) | 4.63 (3.29, 6.67) | 0.193 |
| E2 (pg/mL） | 38.27 (28.93, 52.00) | 37.80 (28.79, 51.09) | 0.840 |
| AMH (ng/mL） | 3.46(1.84, 6.41) | 4.34(2.57, 6.99) | 0.001 |
| Day of blastocyst, n, (%) |  |  | <.001 |
| Day 5 | 225 (31.38) | 492 (68.62) |  |
| Day 6 | 99 (52.38) | 90 (47.62) |  |
| ICM, n(%) |  |  | <.001 |
| A | 61 (24.40) | 189 (75.60) |  |
| B | 243 (38.94) | 381 (61.06) |  |
| C | 20 (62.50) | 12 (37.50) |  |
| TE, n(%) |  |  | <.001 |
| A | 36 (25.53) | 105 (74.47) |  |
| B | 223 (34.15) | 430 (65.85) |  |
| C | 65 (58.04) | 47 (41.96) |  |
| Hatching status, n(%) |  |  | 0.001 |
| Unhatched | 53 (45.69) | 63 (54.31) |  |
| Early hatching | 204 (36.69) | 352 (63.31) |  |
| Late hatching | 50 (25.38) | 147 (74.62) |  |
| Fully hatched | 17 (45.95) | 20 (54.05) |  |
| Type of Infertility, n(%) |  |  | 0.114 |
| Primary | 131 (40.43) | 267 (59.57) |  |
| Secondary | 193 (54.12) | 315 (45.88) |  |

Note: ICM= Inner cell mass; TE =trophectoderm; BMI= body mass index; FSH= follicle-stimulating hormone; LH= luteinizing [hormone](https://medlineplus.gov/hormones.html); E2= estradiol.
