# Supplementary material for: Association between hatching status and pregnancy outcomes in single blastocyst transfers: a retrospective cohort analysis
Source: J Assist Reprod Genet. 2025 Mar 28;42(5):1707–15. doi: 10.1007/s10815-025-03450-4 (PMC12167215; doi:10.1007/s10815-025-03450-4)
Supplement: Supplementary file 3 — Supplementary file3 (DOCX 20 KB) [file 10815_2025_3450_MOESM3_ESM.docx]

| Supplementary table 3 Analysis of factors related to live birth | | | |
| --- | --- | --- | --- |
| Variables | Non-live birth  (n = 443) | Live birth  (n = 463) | *P* |
|  |  |  |  |
| Age of women (years) | 32.56 ± 4.61 | 31.27 ± 4.27 | <.001 |
| BMI (kg/m²） | 24.95 ± 4.47 | 24.52 ± 4.33 | 0.138 |
| Endometrial thickness (mm） | 9.33 ± 1.74 | 9.96 ± 1.80 | <.001 |
| Duration of infertility (years) | 3.00 (1.00, 5.00) | 3.00 (1.00, 5.00) | 0.052 |
| Basal hormone profile |  |  |  |
| FSH (mIU/mL） | 7.03 (6.04, 8.39) | 7.02 (5.86, 8.00) | 0.227 |
| LH (mIU/mL） | 4.44 (3.17, 6.37) | 4.70 (3.29, 6.63) | 0.271 |
| E2 (pg/mL） | 38.27 (29.70, 52.00) | 37.27 (28.00, 51.45) | 0.530 |
| AMH (ng/mL） | 3.62 (1.89, 6.47) | 4.35 (2.78, 7.01) | <.001 |
| Day of blastocyst, n, (%) |  |  | <.001 |
| Day 5 | 322 (44.91) | 395 (55.09) |  |
| Day 6 | 121 (64.02) | 68 (35.98) |  |
| ICM, n(%) |  |  | <.001 |
| A | 97 (38.80) | 153 (61.20) |  |
| B | 323 (51.76) | 301 (48.24) |  |
| C | 23 (71.88) | 9 (28.12) |  |
| TE, n(%) |  |  | <.001 |
| A | 50 (35.46) | 91 (64.54) |  |
| B | 319 (48.85) | 334 (51.15) |  |
| C | 74 (66.07) | 38 (33.93) |  |
| Hatching status, n(%) |  |  | 0.008 |
| Unhatched | 70 (60.34) | 46 (39.66) |  |
| Early hatching | 269 (48.38) | 287 (51.62) |  |
| Late hatching | 82 (41.62) | 115 (58.38) |  |
| Fully hatched | 22 (59.46) | 15 (40.54) |  |
| Type of Infertility, n(%) |  |  | 0.198 |
| Primary | 185 (46.48) | 213 (53.52) |  |
| Secondary | 258 (50.79) | 250 (49.21) |  |

Note: ICM= Inner cell mass; TE =trophectoderm; BMI= body mass index; FSH= follicle-stimulating hormone; LH= luteinizing [hormone](https://medlineplus.gov/hormones.html); E2= estradiol.
