# Supplementary material for: Association between hatching status and pregnancy outcomes in single blastocyst transfers: a retrospective cohort analysis
Source: J Assist Reprod Genet. 2025 Mar 28;42(5):1707–15. doi: 10.1007/s10815-025-03450-4 (PMC12167215; doi:10.1007/s10815-025-03450-4)
Supplement: Supplementary file 4 — Supplementary file4 (DOCX 19 KB) [file 10815_2025_3450_MOESM4_ESM.docx]

| Supplementary table 4 Interaction analysis of hatching status, female age, and embryo quality on clinical pregnancy | | | | | | | |
| --- | --- | --- | --- | --- | --- | --- | --- |
| **Clinical Pregnancy** | | | Unhatched | Early hatching | Late hatching | Fully hatched | *P* |
| **Clinical Pregnancy** | | |  |  |  |  |  |
| Age | | |  |  |  |  | 0.005 |
| < 35^a^ | | N | 61.7% (50/81) | 65.5% (264/403) | 76.0% (111/146) | 65.5% (19/29) |  |
|  | | Adjusted OR  (95%Cl) | Ref | 1.187  (0.708-1.990) | 2.109  (1.142-3.896) | 1.686  (0.666-4.265) |  |
| ≥35^b^ | | N | 38.9% (14/36) | 57.5% (88/153) | 70.6% (36/51) | 12.5% (1/8) |  |
|  | | Adjusted OR  (95%Cl) | Ref | 1.915  (0.834-4.397) | 3.964  (1.466-10.732) | 0.234  (0.022-2.459) |  |
| Blastocyst quality | | |  |  |  |  | 0.198 |
| Good^c^ | N | | 63.4% (59/93) | 66.9% (316/472) | 77.3% (133/172) | 65.2% (15/23) |  |
|  | Adjusted OR  (95%Cl) | | Ref | 1.224  （0.757-1.979） | 2.305  （1.295-4.102） | 1.609  （0.593-4.364） |  |
| Poor^d^ | N | | 20.8% (5/24) | 42.9% (36/84) | 56.0% (14/25) | 35.7% (5/14) |  |
|  | Adjusted OR  (95%Cl) | | Ref | 2.604  （0.864-7.850） | 4.317  （1.183-15.752） | 1.759  （0.385-8.033） |  |

a adjusted for **endometrium thickness, blastocyst quality,** embryo cryopreservation duration**, day of blastocyst**

b adjusted for **endometrium thickness, AMH, blastocyst quality,** year of Infertility, **day of blastocyst**

c adjusted for **endometrium thickness, female age,** embryo cryopreservation duration, **day of blastocyst**

d adjusted for **female age**
