# Supplementary material for: Association between hatching status and pregnancy outcomes in single blastocyst transfers: a retrospective cohort analysis
Source: J Assist Reprod Genet. 2025 Mar 28;42(5):1707–15. doi: 10.1007/s10815-025-03450-4 (PMC12167215; doi:10.1007/s10815-025-03450-4)
Supplement: Supplementary file 5 — Supplementary file5 (DOCX 20 KB) [file 10815_2025_3450_MOESM5_ESM.docx]

| Supplementary table 5 The clinical outcomes of each group among different protocols | | | | | |
| --- | --- | --- | --- | --- | --- |
| Live birth | Unhatched | Early hatching | Late hatching | Fully hatched | *P* |
| Natural cycle | 53.3%(8/15) | 52.7%(39/74) | 60.6%(20/33) | 37.5%(3/8) | 0.681 |
| Ovulation induction cycle | 50.0%(3/6) | 53.8%(21/39) | 37.5%(3/8) | 75.0%(3/4) | 0.691 |
| Artificial cycle | 36.5%(35/96) | 51.2%(227/443) | 59.00%(92/156) | 36.0%(9/25) | 0.003 |

| Clinical pregnancy | Unhatched | Early hatching | Late hatching | Fully hatched | *P* |
| --- | --- | --- | --- | --- | --- |
| Natural cycle | 66.7%（10/15） | 56.8%(42/74) | 90.9%(30/33) | 50.0%(4/8) | 0.002 |
| Ovulation induction cycle | 50.0%（3/6) | 61.5%(24/39) | 50.0%(4/8) | 75.0%(3/4) | 0.809 |
| Artificial cycle | 46.9%(45/96) | 64.6%(286/443) | 72.4%(113/156) | 52.0%(13/25) | 0.010 |

| Live birth | Unhatched | Early hatching | Late hatching | Fully hatched | *P* |
| --- | --- | --- | --- | --- | --- |
| Non-Artificial cycle | 52.38%(11/21) | 53.10%(60/113) | 56.10%(23/41) | 50%(6/12) | 0.979 |
| Artificial cycle | 36.5%(35/96) | 51.2%(227/443) | 59.00%(92/156)^a^ | 36.0%(9/25) ^b^ | 0.003 |
|  |  |  |  |  |  |
| Clinical pregnancy | Unhatched | Early hatching | Late hatching | Fully hatched | *P* |
| Non-artificial cycle | 61.9%(13/21)） | 58.41%(66/113) | 82.93%(34/41) | 58.33%(7/12) | 0.043 |
| Artificial cycle | 46.9%(45/96)^b^ | 64.6%(286/443) | 72.4%(113/156)^a^ | 52.0%(13/25) | 0.010 |
