# Supplementary material for: Association between hatching status and pregnancy outcomes in single blastocyst transfers: a retrospective cohort analysis
Source: J Assist Reprod Genet. 2025 Mar 28;42(5):1707–15. doi: 10.1007/s10815-025-03450-4 (PMC12167215; doi:10.1007/s10815-025-03450-4)
Supplement: Supplementary file 6 — Supplementary file6 (DOCX 17 KB) [file 10815_2025_3450_MOESM6_ESM.docx]

| Supplementary table 6 The clinical outcomes observed in each group at Day 5 of gestation. | | | | | |
| --- | --- | --- | --- | --- | --- |
| D5 | Unhatched | Early hatching | Late hatching | Fully hatched | *P* |
| Clinical pregnancy | 65.55%(59/90) | 67.11%(306/456) | 75.66%(115/152) | 63.16%(12/19) | 0.200 |
| Live birth | 50.0%(45/90) | 53.95%(246/456) | 61.18%(93/152) | 57.89%(11/19) | 0.318 |
