# Supplementary material for: Association between hatching status and pregnancy outcomes in single blastocyst transfers: a retrospective cohort analysis
Source: J Assist Reprod Genet. 2025 Mar 28;42(5):1707–15. doi: 10.1007/s10815-025-03450-4 (PMC12167215; doi:10.1007/s10815-025-03450-4)
Supplement: Supplementary file 7 — Supplementary file7 (DOCX 18 KB) [file 10815_2025_3450_MOESM7_ESM.docx]

| Supplementary table 7 Clinical outcomes in each group under different fertilization methods | | | | | |
| --- | --- | --- | --- | --- | --- |
|  | IVF | | | | *P* |
| Clinical Outcome | Unhatched  n=91 | Early hatching  n=405 | Late hatching  n=138 | Fully hatched  n=26 |  |
| Clinical pregnancy | 48 (52.75)^a^ | 263 (64.94) | 104 (75.36)^b^ | 15 (57.69) | 0.004 |
| Live birth | 34 (37.36)^a^ | 215 (53.08) | 84 (60.87)^b^ | 11 (44.53) | 0.004 |
|  | ICSI | | | | *P* |
| Clinical Outcome | Unhatched  n=26 | Early hatching  n=151 | Late hatching  n=59 | Fully hatched  n=11 |  |
| Clinical pregnancy | 16 (61.54) | 90 (59.60) | 43 (72.88) | 5 (45.45) | 0.202 |
| Live birth | 12 (46.15) | 72 (47.68) | 31 (52.54) | 4 (36.36) | 0.772 |
